# Mitochondrial Side Effects of Surgical Prophylactic Antibiotics Ceftriaxone and Rifaximin Lead to Bowel Mucosal Damage

**DOI:** 10.3390/ijms23095064

**Published:** 2022-05-03

**Authors:** Bálint Baráth, Dávid K. Jász, Tamara Horváth, Bence Baráth, Gergely Maróti, Gerda Strifler, Gabriella Varga, Lilla Sándor, Domonkos Perényi, Szabolcs Tallósy, Tibor Donka, Péter Jávor, Mihály Boros, Petra Hartmann

**Affiliations:** 1 Institute of Surgical Research, University of Szeged, 6724 Szeged, Hungary; barath.balint@med.u-szeged.hu (B.B.); jasz.david.kurszan@med.u-szeged.hu (D.K.J.); horvath.tamara@med.u-szeged.hu (T.H.); gira86@gmail.com (G.S.); varga.gabriella.1@med.u-szeged.hu (G.V.); san.lilla97@gmail.com (L.S.); pdodo07@gmail.com (D.P.); tallosy.szabolcs@med.u-szeged.hu (S.T.); tibor.donka96@gmail.com (T.D.); boros.mihaly@med.u-szeged.hu (M.B.); 2 Department of Traumatology, University of Szeged, 6725 Szeged, Hungary; javorpeter25@gmail.com; 3 Department of Pathology, University of Szeged, 6725 Szeged, Hungary; barath.bence@med.u-szeged.hu; 4 Institute of Plant Biology, Biological Research Center, ELKH, 6726 Szeged, Hungary; maroti.gergely@brc.hu

**Keywords:** mitochondrial dysfunction, surgical prophylaxis, antibiotics, bowel microbiome, ceftriaxone, rifaximin

## Abstract

Despite their clinical effectiveness, a growing body of evidence has shown that many classes of antibiotics lead to mitochondrial dysfunction. Ceftriaxone and Rifaximin are first choice perioperative antibiotics in gastrointestinal surgery targeting fundamental processes of intestinal bacteria; however, may also have negative consequences for the host cells. In this study, we investigated their direct effect on mitochondrial functions in vitro, together with their impact on ileum, colon and liver tissue. Additionally, their impact on the gastrointestinal microbiome was studied in vivo, in a rat model. Rifaximin significantly impaired the oxidative phosphorylation capacity (OxPhos) and leak respiration in the ileal mucosa, in line with increased oxidative tissue damage and histological changes following treatment. Ceftriaxone prophylaxis led to similar changes in the colon mucosa. The composition and diversity of bacterial communities differed extensively in response to antibiotic pre-treatment. However, the relative abundances of the toxin producing species were not increased. We have confirmed the harmful effects of prophylactic doses of Rifaximin and Ceftriaxone on the intestinal mucosa and that these effects were related to the mitochondrial dysfunction. These experiments raise awareness of mitochondrial side effects of these antibiotics that may be of clinical importance when evaluating their adverse effects on bowel mucosa.

## 1. Introduction

Several classes of antibiotics, despite their clinical effectiveness, have been shown to lead to severe mitochondrial dysfunction in eukaryotes [1,2,3]. The mechanism by which antibiotics damage mitochondria in the host is the impairment of conserved biochemical processes originating from bacterial progenitors [4,5]. These include disruption of mitochondrial electron transport chain (ETC) function leading to the production of lethal reactive oxygen species (ROS) [6], altering ion permeability of the mitochondrial membrane [7] and inhibiting oxidative phosphorylation by the decoupling of the F0 and F1 factors of mitochondrial ATPase [8]. Certain antibiotics are known to inhibit mitochondrial biogenesis through the large (39S) and small ribosome (28S) and as a consequence, the mitochondrial protein translation [9,10]. These “off-target” effects of antibiotics can also be beneficial according to a recent clinical trial testing antibiotics in anticancer therapy [11]. Yet, the possible role of antibiotics in mitochondrial dysfunction is unclear and seems to be tissue- and cell line-dependent [12].

Surgical site infections (SSI) and associated morbidity still have high incidence in colorectal surgery due to the high intraluminal bacterial load [13]; therefore, guidelines recommend mechanical bowel preparation and oral antibiotic pre-treatment combined with parenteral surgical antibiotic prophylaxis (SAP) before the intervention [14]. However, the use of Cephalosporins, the first line antibiotics for prevention has been linked to considerable adverse effects [15,16], which may occur during prolonged treatment [17,18], but can also be triggered by a single dose [16,19]. The tissue toxicity of Cephalosporins is closely related to the inhibition of mitochondrial substrate uptake and the enhancement of mitochondrial-derived ROS production [20,21]. Cephalosporins are often combined with Rifamycin derivatives used in the treatment of infectious diseases in humans and domestic animals [22,23]. However, available data indicate that Rifamycins affect the viability of eukaryotic cells and cause various pathological changes in them by inhibiting mitochondrial RNA polymerase [24] and promoting the inhibition of ATP synthesis [25]. Whether each combination of antibiotics can enhance or counteract the mitochondrial side effects of the other has not yet been investigated.

We also need to emphasize the impact of antibiotics on the microbiome, as a consequence of their complex effects on bowel toxicity and eventually on mitochondria. Prophylactic antibiotics reduce the total bacterial count (mainly anaerobes), and change the composition of microflora [26], primarily in the large intestines, with potential adverse effects for the host [27,28,29]. Emerging evidence suggests that gut microbiota signal to the mitochondria of mucosal cells, including epithelial cells and immune cells, and dysbiosis may be associated with intestinal inflammation [30].

Our aim in the present study was to characterize the mechanistic effects of clinically relevant levels of Ceftriaxone, a third-generation cephalosporin, and the poorly absorbed Rifamycin-derivative, Rifaximin on mammalian cells, both in vitro and in vivo. Previous studies have shown that mitochondria in mammalian cells can be damaged by antibiotic treatment, but these results were demonstrated on either in vitro cell cultures [9,12,31,32,33,34,35] or at concentrations considerably higher than those applied clinically [36,37]. Mitochondrial functional measurements from fresh biopsy samples and the integrated analysis of mitochondria-related mucosal cell toxicity and the gut microbiota composition were not performed. Here, we showed that these drugs commonly used in combination prior to colorectal surgery caused mitochondrial dysfunction and ROS overproduction in mammalian cells, ultimately leading to the accumulation of oxidative tissue damage. We found that these deleterious effects on the bowel mucosa were independent of the composition of the microbiome. These results raise the possibility of preventing or diminishing the adverse effects of these antibiotics with protective agents targeting mitochondria.

## 2. Results

### 2.1. In Vitro Experiments

**Figure 1:**
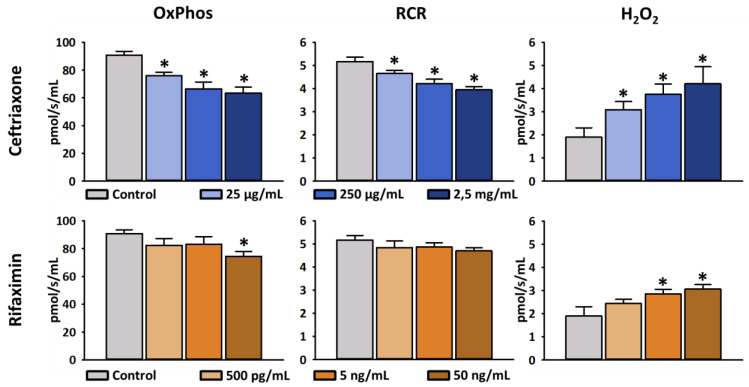
Effects of Ceftriaxone and Rifaximin on oxidative phosphorylation (OxPhos), respiratory acceptor control ratio (RCR) and hydrogen-peroxide (H_2_O_2_) production of isolated mitochondria. Concentrations of antibiotics are based on their peak plasma concentration following the administration of a single dose (250 µg/mL Ceftriaxone or 5 ng/mL Rifaximin) and tenfold higher or lower concentrations for comparison. Darker shades indicate higher concentrations. Blue columns: Ceftriaxone treatment; Orange columns: Rifaximin treatment; Data are presented as mean ± SEM. * *p* < 0.05 vs. control (one-way ANOVA, Holm–Sidak test) are considered as significant.

Firstly, an in vitro pilot study was conducted on isolated mitochondria in order to verify the effects of Rifaximin, Ceftriaxone and their combination on the mitochondrial respiration and H_2_O_2_ production, using high (100 µg/mL and an order of magnitude higher and lower) concentrations. As a result, high concentrations had a toxic effect on mitochondria leading to completely abolished mitochondrial respiration and non-functioning mitochondria (data not shown). Thereafter, the experiment was repeated using the expected peak plasma concentrations of antibiotics after their *per os* and *intraperitoneal* therapeutic use [38,39], and at concentrations ten times higher or lower for comparison (Figure 1). We found that Ceftriaxone dose dependently impairs mitochondrial OxPhos (76.0 ± 2.5 pmol/s/mL, 66.5 ± 5 pmol/s/mL and 63.5 ± 4.2 pmol/s/mL following 25 µg/mL, 250 µg/mL and 2500 µg/mL Ceftriaxone treatment, respectively) as compared to the controls (90.8 ± 2.7 pmol/s/mL). RCR was decreased at the therapeutic concentration of Ceftriaxone (250 µg/mL) vs. control (4.2 ± 0.2 pmol/s/mL vs. 5.2 ± 0.2). H_2_O_2_ production showed a significant increase at the therapeutic concentration of Ceftriaxone as compared to control mitochondria (3.8 ± 0.4 pmol/s/mL vs. 1.9 ± 0.4 pmol/s/mL). The mitochondrial OxPhos and RCR tended to decrease following Rifaximin treatment, but no significant changes could be detected using therapeutic doses; however, therapeutic Rifaximin concentration (5 ng/mL) resulted in a significant increase in mitochondrial H_2_O_2_ production (2.9 ± 0.2 pmol/s/mL vs. control 1.9 ± 0.4 pmol/s/mL). The relationship between mitochondrial OxPhos, RCR and H_2_O_2_ production was also investigated within the Ceftriaxone and Rifaximin groups, separately. Within the Ceftriaxone group we found a strong correlation between each parameter (Pearson’s R > 0.9). While in the Rifaximin group we saw a strong correlation between OxPhos and RCR, as well as between OxPhos and H_2_O_2_ (Pearson’s R > 0.7), the correlation between H_2_O_2_ and RCR was slightly weaker (Pearson’s R = 0.695).

### 2.2. In Vivo Experiments

#### 2.2.1. Mitochondrial Respiration

Our protocol provides an opportunity to analyze the activity of mitochondria at different respiratory states in different tissues. First, the electron transfer pathway that supports the succinate-linked respiration rate (complex II-linked state II respiration) was determined in the liver, ileum and colon mitochondria. For this purpose, complex I function was blocked using rotenone and complex II was stimulated with a succinate substrate. The OxPhos (state III) was determined after adding saturating ADP to fuel ATP synthase activity. Then, the coupling efficiency of the mitochondria was determined by measuring the respiration rate in the presence of an ATP synthase inhibitor oligomycin (state IV). This state is also called the leak state when respiration is maintained by protons leaking through the inner membrane independently of ATP synthase.

**Figure 2:**
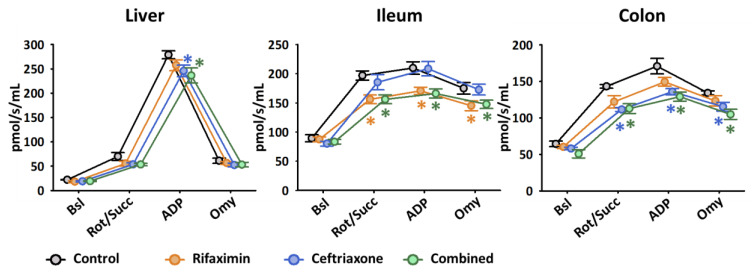
Mitochondrial respiratory function in the liver, terminal ileum and distal colon following systemic antibiotic treatment. After the registration of baseline (Bsl) respiratory activity, state II respiration was measured in the presence of rotenone and succinate (Rot/Succ), OxPhos was measured after the addition of adenosine diphosphate (ADP), and leak respiration in the presence of oligomycin (Omy). Rifaximin group is marked with orange, Ceftriaxone group is marked with blue, combined treatment is marked with green, while control group is marked with grey symbols. Data are presented as mean ± SEM. * *p* < 0.05 vs. control (one-way ANOVA, Holm–Sidak test) are considered as significant.

Consistent with the in vitro results, Ceftriaxone and the combined treatments significantly decreased OxPhos as compared to untreated controls (245.9 ± 11.9 pmol/s/mL and 236.5 ± 15.3 pmol/s/mL vs. 279.0 ± 8.3 pmol/s/mL), while Rifaximin had no significant effect in the liver mitochondria (Figure 2).

Rifaximin significantly deteriorated the mitochondrial respiration in succinate-linked (156.1 ± 7.4 pmol/s/mL vs. 197.0 ± 7.9 pmol/s/mL), OxPhos (170.8 ± 6.1 pmol/s/mL vs. 209.8 ± 10.8 pmol/s/mL) and leak states (145.3 ± 8.5 pmol/s/mL vs. 175.1 ± 9.8 pmol/s/mL) in the terminal ileum as compared to controls. Combined treatment caused parallel respiratory changes in the ileum.

No significant change was detected in the mitochondrial respiratory states in the ileum samples following Ceftriaxone treatments. Alternatively, there was a significant decrease in the colon in response to Ceftriaxone (85.8 ± 2.1 pmol/s/mL vs. 110.5 ± 6.4 pmol/s/mL in state II, 135.9 ± 4.2 pmol/s/mL vs. 170.9 ± 10.6 pmol/s/mL in OxPhos and 115.5 ± 6.1 pmol/s/mL vs. 134.3 ± 2.9 pmol/s/mL in leak state vs. control) and combined treatments in each state.

#### 2.2.2. Biochemical Parameters

**Figure 3:**
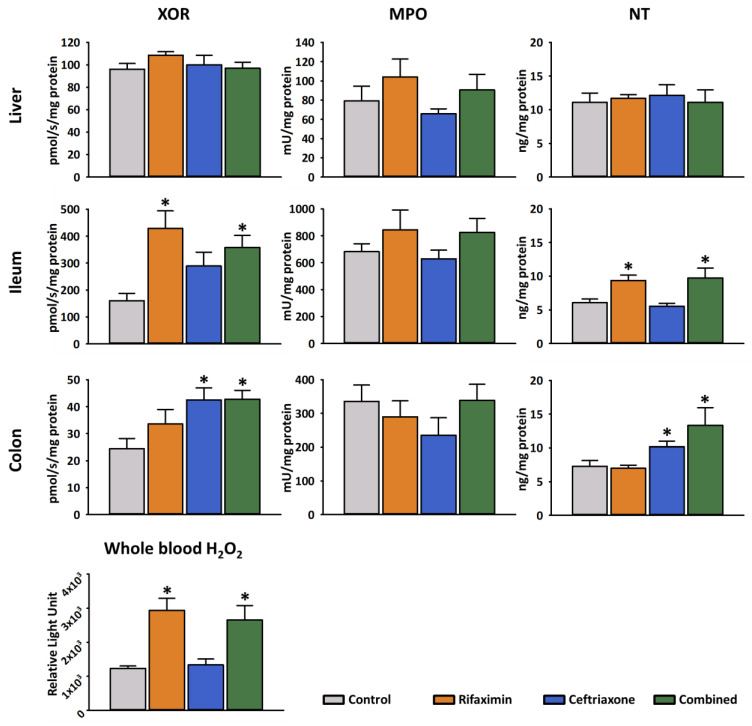
Oxido-reductive stress markers in different tissues following systemic antibiotic treatment. Tissue xanthine oxidoreductase (XOR) activity, myeloperoxidase (MPO) activity and nitrotyrosine (NT) levels were measured in the liver, the terminal ileum and the distal colon, and whole blood hydrogen-peroxide (H_2_O_2_) production was determined following 3 days of antibiotic treatment. Orange columns: Rifaximin treatment; Blue columns: Ceftriaxone treatment; Green columns: combined treatment. Data are presented as mean ± SEM. * *p* < 0.05 vs. control (one-way ANOVA, Holm–Sidak test) are considered as significant.

Tissue XOR-, MPO activity, and NT content was measured from liver, terminal ileum and distal colon homogenates. XOR is a key enzyme in purine catabolism, and also catalyzes the reduction of nitrates and nitrites into nitric oxide (NO). During this process, ROS are produced, which can be deleterious to the cells. As expected, a significant increase was detected in the ileal XOR activity following Rifaximin and combined treatment, while XOR activity in the colon was elevated only in the Ceftriaxone and combined groups (Figure 3). NT formation is a marker of nitrosative stress within the tissues and correlates with peroxynitrite production. NT content significantly increased in the ileum in the groups that received Rifaximin or combined treatment and in the colon following Ceftriaxone and combined treatment. MPO is produced mostly by PMN leukocytes upon their activation. There was no significant difference in MPO activity in response to antibiotic treatments in either part of the intestines. No significant changes could be measured in the liver in any parameters. The hydrogen-peroxide levels in the whole blood were significantly higher in the Ceftriaxone and combined groups following the 3-day treatment protocol when compared to samples from control animals (Figure 3). Among serum necroenzymes, LDH showed a significant increase in response to any antibiotic pretreatment (Table 1).

#### 2.2.3. Histopathology (CLSEM, H&E and PAS Staining)

**Figure 4:**
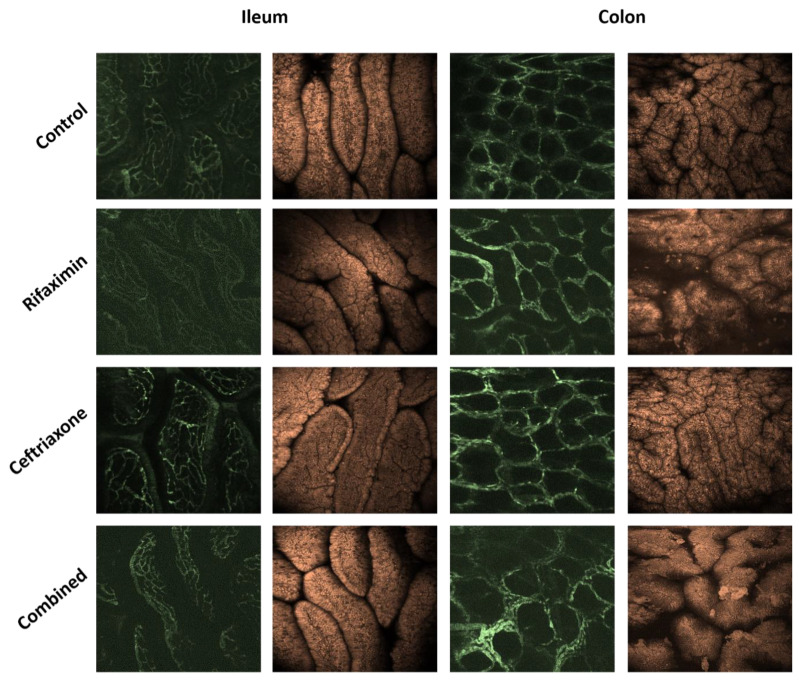
In vivo histology of the luminal surface of the small intestine and colon using CLSM. Microvascular structures were visualized with *i.v.* FITC-dextran and the mucosal surface with topical acriflavine dye.

The morphological changes in the left liver, in the small intestinal villi and distal colon mucosa were evaluated by means of in vivo imaging, using CLSEM. The microvessels were visualized with FITC-dextran administration, while the morphological changes were examined with topical acriflavine staining. In the case of the terminal ileum, antibiotic treatments including Ceftriaxone or Rifaximin or their combinations did not result in damage to the microvasculature or the mucosal surface of villi as compared to the control animals. Similarly, following Ceftriaxone treatment, the colon mucosa and microvasculature showed normal morphology. However, Rifaximin and combined treatments remarkably deranged the epithelial morphology in the colon mucosa. The observed changes included enlarged interglandular space and shedding of epithelial cells. Moreover, the degree of epithelial shedding was more prominent after combined treatment than in the Rifaximin group (Figure 4).

**Figure 5:**
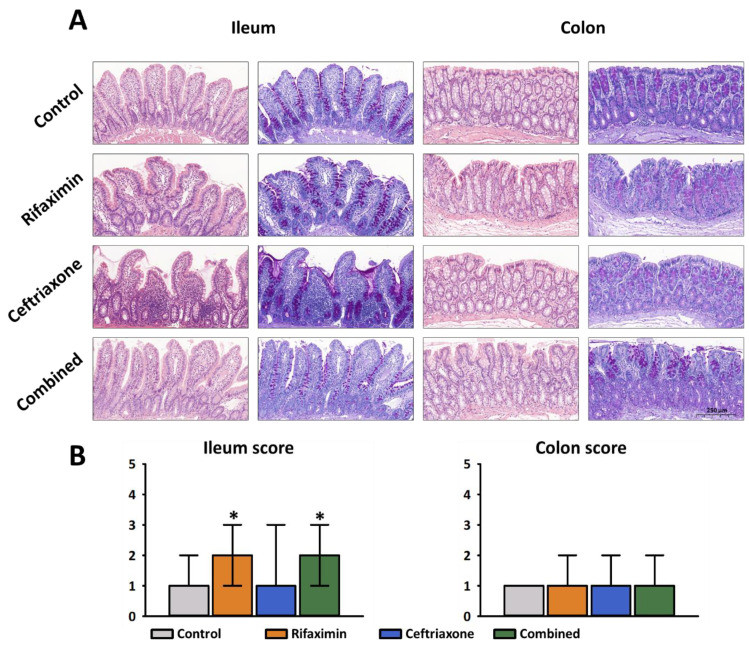
Histopathology. (**A**) Representative photomicrographs of the examined groups. Images were taken with a 5× objective after hematoxylin and eosin (H&E) and periodic acid-schiff (PAS) staining of the biopsy samples. (**B**) Histopathological evaluation of mucosal damage in the terminal ileum and distal colon. Semi-quantitative scoring system (0–5 grade) was used representing a composite of number of villous atrophy, cellular infiltration, apoptosis and tissue oedema formation. Orange columns: Rifaximin treatment; Blue columns: Ceftriaxone treatment; Green columns: combined treatment. Data are presented as median and minimum/maximum values. * *p* < 0.05 vs. control (ANOVA on Ranks, Tukey’s test) are considered as significant.

The degree of intestinal injuries in the examined groups were demonstrated in representative histologic images (Figure 5). Semi-quantitative estimation of the mucosal damage revealed normal histology in the control group and biopsy samples taken from untreated animals had a median grade of injury of 1 in the ileum and colon, respectively. Rifaximin treatment induced significant damage and resulted in ileal mucosal injury of a median grade of 2, whereas Ceftriaxone treatment resulted in a statistically non-significant deterioration in the colon mucosa with a median grade of 1 (Figure 5).

#### 2.2.4. Analysis of the Bowel Microbiome

**Figure 6:**
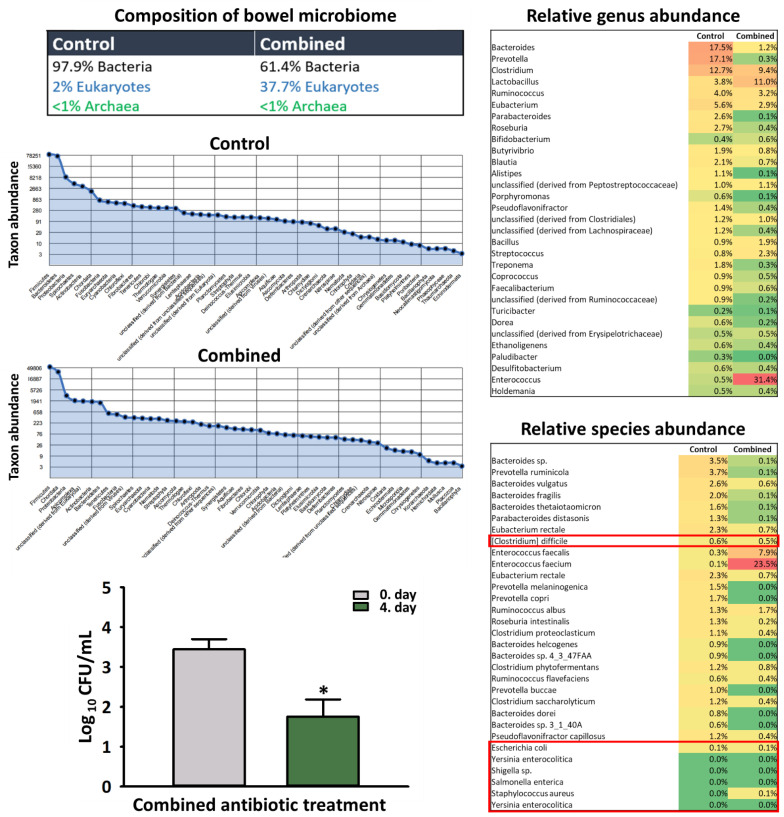
Qualitative and quantitative analysis of the bowel microbiome. The number of viable anaerobic bacteria was reduced with 2 orders of magnitude after 3-day-long antibiotic pretreatment (expressed as Log **_10_** CFU/mL). Diversity and abundance of the bowel microbiome have changed in response to combined antibiotic treatment. Metagenomic sequencing did not indicate the overgrowth of toxin-producing bacteria such as *C. difficile*, *E. coli*, *K. oxytoca*, *Y. enterocolitica*, *S. aureus*, *S. enterica* and Shigellae. Black column: Day 0 (before treatment); Gray columns: 4th day (after combined treatment). Data are presented as mean ± SEM. * *p* < 0.05 vs. control (one-way ANOVA, Holm–Sidak test) are considered as significant.

The microbiological plating method revealed a two-magnitude of diminution in the number of bacterial colonies following combined antibiotic treatment (Figure 6). The abundance and composition of the bacterial flora changed significantly following combined antibiotic treatment. The relative abundance of certain *Enterococci* species (*E. faecium*, *E. faecalis*) increased significantly, while the presence of toxin producing and facultative pathogenic bacteria, such as *C. difficile*, *E. coli*, *K. oxytoca* and *Y. enterocolitica* did not change or even diminished.

## 3. Discussion

The assumption that antibiotics are effective against bacteria while not harming the host is based on the belief that they only act on components found specifically in bacteria. Among the surgical prophylactic antibiotics, Ceftriaxone has high affinity for penicillin binding proteins and inhibits bacterial cell wall biosynthesis, while Rifaximin binds to the β subunit of the bacterial DNA-dependent RNA polymerase–all these cellular constituents are unique for prokaryotes [38,40]. Both antibiotics are often used as monotherapy and in combination prior to surgical procedures, either as a single dose or as a pre-treatment for 1–3 days [16,19,22,23]. They have an undoubted benefit in reducing the incidence of SSI, however, the toxicity of these antibiotics to the eukaryotic organism has been widely demonstrated [41]. Thus, SAP with Ceftriaxone and Rifaximin carries the potential risk that antibiotic-induced tissue damage will be added to the intestinal stress caused by the disease and the surgical procedure, and in addition to negatively affecting the outcome of the treatment, can lead to serious side effects.

The pathogenic role of mitochondrial dysfunction has been shown to underlie the tissue toxicity of several antibiotics of the cephalosporin class and Rifamycin derivatives. It was previously described that Rifampicin inhibits mitochondrial mRNA synthesis in isolated mitochondria, leading to the disruption of the inner membrane of the mitochondria as a result of decreased protein production [24,25]. Furthermore, first generation cephalosporins Cephaloridine and Cephaloglycin were found to negatively impact substrate uptake, mainly succinate in isolated mitochondria and rabbits. This effect is caused by the acylation of membrane transport proteins [20,21]. However, the effects of Ceftriaxone and Rifaximin and the consequences of their combination at prophylactic concentrations on intestinal mucosal mitochondrial functions have not yet been investigated. Therefore, our study further advances the understanding of antibiotic action in mammalian systems on mitochondria. The investigations were carried out with high-resolution respirometry, which is a robust tool for the assessment of mitochondrial functions in the bowel mucosa, because it provides immediate results on the elements of the mitochondrial respiratory chain without pre-treatment or fixation of biopsy samples [42,43]. Our primary finding is that mitochondrial dysfunction is caused by clinically relevant doses of antibiotics, both in cell culture and in vivo, using experimental animals (rats). These results are in accordance with previous in vitro studies, in which bactericidal antibiotics induce a common oxidative damage pathway [4,44]. In the present study, mitochondrial toxicity was dose-dependent and, nevertheless, proved to be non-specific in its mechanism. Both Ceftriaxone and Rifaximin inhibited OxPhos, decreased RCR and increased ROS production in vitro. This was further confirmed by our in vivo experiments, in which these antibiotics not only inhibited the OxPhos, but the succinate uptake and the ETC coupling efficiency as well. A further finding of our study is that, contrary to preliminary concerns, the mitochondrial damaging effects of these antibiotics when used in combination were not additive as they did not enhance the destruction in OxPhos and mitochondrial respiration coupling.

Bactericidal antibiotics are generally considered to exert tissue damage due to the related accumulation of oxidative stress in tissues [4,44], however, there are many cases that prove otherwise, as it has been shown for Ceftriaxone. In parallel with oxidative effects, Ceftriaxone also upregulates endogenous antioxidant enzymes in the nervous tissue, which eventually makes it a neuroprotective drug [45]. Rifaximin has been described to prevent the progression of steatohepatitis by downregulating β-oxidation and modulation of the small intestinal microbiome [46]. In this study we found that while rifaximin caused oxidative damage in the small intestine, ceftriaxone caused oxidative damage mainly in the large intestine, as indicated by increased XOR activity and NT levels. These changes are unlikely to be related to inflammatory cellular infiltration, as evidenced neither by histology nor by the nearly unchanged level of MPO activity. The above detailed respirometry data suggest a likely role for mitochondrial-derived free radical production on the background of oxidative tissue damage.

All antibiotic-treated groups showed evidence of minor mucosal damage following the 3-day treatment protocol. These changes were more pronounced following Rifaximin treatment, especially in the terminal ileum. Rifaximin is administered orally and absorbed poorly (<1%), therefore it reaches a high intraluminal concentration. It has been previously described that Rifaximin solubility increases in the presence of bile acids [47]. In our findings Rifaximin affected the terminal ileum but not as much the colon. This could be a result of the enterohepatic recirculation and the lower solubility of the drug in the colon. Interestingly, the H_2_O_2_ levels of the whole blood increased only in the case of Rifaximin treatment, even though a very small percentage of Rifaximin is absorbed into the circulation, unlike Ceftriaxone, which is administered intravenously. This is due to the mucosal damage induced by Rifaximin being present over an extensive area of the ileum, which may explain the elevated H_2_O_2_ levels of the whole blood. In case of Ceftriaxone the mucosal changes were less pronounced. Ceftriaxone is parenterally administered and has a high protein-binding ability resulting in slow biliary excretion, reaching lower intraluminal concentration [48,49]. Roughly one third of the administered drug is eliminated through the bile, in a microbiologically inactive form. The lesions are likely a result of the prolonged elimination and thus longer direct exposition to the drug.

Consistent with previous data, we observed two orders of magnitude changes in the number of bacteria and a shift in the composition of the bowel microbiome as a response to antibiotic pre-treatment [50,51]. Of note, the overgrowth or the increase in the relative proportion of pathogens associated with gastrointestinal toxicity, exemplified by *Clostridium difficile* were not confirmed. In the case of short-term antibiotic treatment, most bacterial groups recovered after treatment, but several taxa did not (even after 14 days) [52], thereby affecting host physiology and potentially host health [53]. Nonetheless, the importance of such disturbance of the microbiota by prophylactic antibiotics in postoperative care is largely unknown and require further investigation.

In the present study no chronic nor “single-shot” efficacy studies have been performed with this antibiotic combination. Furthermore, it is important to mention that we have only used animal models in our studies. We have not tested the effects of antibiotics in a clinical setting.

Herein, we have confirmed the harmful effects of prophylactic doses of surgical prophylactic antibiotics Rifaximin and Ceftriaxone on the bowel mucosa in a rat model. We have also demonstrated that these antibiotics cause mitochondrial impairment in the epithelial cells, which is dose-dependent in isolated mitochondria. Mucosal damage was in relation with the mitochondrial dysfunction and oxidative tissue damage in the bowel mucosa. However, was independent of the changes in the bacterial microbiome. These experiments disclose previously unknown, clinically relevant side effects of these antibiotics that should be taken into account in premedication protocols for colorectal surgery.

## 4. Materials and Methods

### 4.1. Study Design

The objective of our work was to investigate the mitochondrial effects of clinically relevant doses of antibiotics used in surgical prophylaxis on mammalian systems in vitro and in vivo.

The experiments were carried out on male Sprague–Dawley rats (n = 72, average weight: 300 ± 25 g, 8–9 weeks of age) housed in an environmentally controlled room with a 12-h light-dark cycle, and kept on commercial rat chow (Standard rat chow LT/n; Innovo Kft, Gödöllő, Hungary) and tap water ad libitum. The experimental protocol was in accordance with EU directive 2010/63 for the protection of animals used for scientific purposes, and it was approved by the National Scientific Ethical Committee on Animal Experimentation (National Competent Authority) with the license number V./1416/2021. This study also complied with the criteria of the US National Institutes of Health Guidelines for the Care and Use of Laboratory Animals.

We performed in vitro experiments to detect the concentration dependent changes in the respiratory activity of isolated mitochondria in response to incubation with antibiotics (Ceftriaxone, Rifaximin or in combination) using high-resolution respirometry (HRR) (Oxygraph-2k, Oroboros Instruments, Innsbruck, Austria). In this experimental series, animals were anesthetized for harvesting biopsy samples using ketamine (45.7 mg/kg *i.p.*) and xylazine (9.12 mg/kg *i.p.*). Then, mitochondria were isolated from the livers of the animals as described by Gnaiger [54]. Isolated mitochondria were incubated with antibiotics at therapeutic serum concentrations [55,56,57] and an order of magnitude higher and lower concentrations, as follows: Ceftriaxone (Ceftriaxon Kabi, Fresenius Kabi Deutschland GmbH, Bad Homburg vor der Höhe, Germany): 25–250–2500 µg/mL, Rifaximin (Normix, Alfasigma S.p.A., Bologna, Italy): 0.5–5–50 ng/mL), for 1 h (n = 12). Subsequently, mitochondria were subjected to HRR to determine mitochondrial oxygen consumption and hydrogen peroxide (H_2_O_2_) production.

In the in vivo experimental series, animals were pre-treated twice daily with the prophylactic dose of Ceftriaxone (15 mg/kg *i.p.*, n = 12) or Rifaximin (8 mg/kg *p.o.* with gavage, n = 12) or the combination of both (n = 12) for 3 days, while control animals received a saline vehicle [57,58,59]. At the end of pre-treatment, animals were anesthetized with sodium pentobarbital (45 mg/kg *i.p.*) for in vivo histology with fluorescence confocal laser scanning endomicroscopy (CLSEM, Five1, Optiscan Pty. Ltd., Melbourne, Australia, excitation wavelength 488 nm; emission detected at 505–585 nm), or tissue samples were taken from the liver, terminal ileum and colon for HRR and biochemical assays. Additionally, stool samples were taken prior to the antibiotic treatment and after the completion of the treatment protocol for proteomics and microbiological analysis. At the end of the experiments, the animals were over-anesthetized with a single overdose of ketamine and xylazine (Figure S1).

### 4.2. Mitochondrial Functional Measurements

#### 4.2.1. Respirometry

The detection chambers of the respirometer were calibrated to 200 nmol/mL oxygen concentration in room air using 2.2 mL Mir05 respiration medium (EGTA 0.5 mM; MgCl_2_ 3 mM; lactobionic acid 60 mM; taurine 20 mM; KH_2_PO_4_ 10 mM; HEPES 20 mM; D-sucrose 110 mM; BSA 1 g/L). In order to determine the mitochondrial respiration of isolated liver mitochondria (in vitro studies) and tissue homogenates (liver, terminal ileum or distal colon from in vivo studies), samples (20–20 µL, respectively) were weighed into the detection chambers. The respiration was investigated using a leak protocol: after registration of baseline respiration, 0.5 μM complex I inhibitor rotenone and 10 mM succinate were added to determine mitochondrial respiratory complex II-linked state II respiration rate of ETC. Then, the oxidative phosphorylation (OxPhos) was investigated by adding saturating concentration of 2.5 mM ADP (complex II-linked state III respiration) to the chamber. Finally, the leak respiration was measured in the leak state by inhibition of ATP synthase after administration of 0.5 μM oligomycin to the medium (state IV respiration). Respiratory acceptor control ratio (RCR) is expressed as the OxPhos/leak ratio and it refers directly to the physiological function of the ETC as the OxPhos-coupling efficiency. The measurements were performed in duplicates and were normalized for protein content. Protein content of the samples was determined with Lowry’s method [60].

#### 4.2.2. Fluorometry

The mitochondrial H_2_O_2_ release, as a marker of reactive oxygen species (ROS) production was monitored fluorimetrically with the Amplex Red/horseradish peroxidase system (AmpR/HRP, Invitrogen, Waltham, MA, USA), whereby AmpR (non-fluorescent) is oxidized to the fluorescent compound, Resorufin (excitation wavelength 563 nm, emission wavelength 587 nm). The chemifluorometric response was stimulated and simultaneously measured with a Smart Fluo-Sensor Green (Oroboros Instruments, Innsbruck, Austria) module (dominant wavelength 525 nm) of the Oxygraph-2k. Calibration of H_2_O_2_ production was performed by known amounts of H_2_O_2_. In this setup, ROS release was measured by using a leak protocol as described in Respirometry.

#### 4.2.3. Tissue Xanthine Oxidoreductase (XOR) Activity

Tissue biopsies were homogenized in phosphate buffer (pH 7.4) containing 50 mM Tris-HCl, 0.1 mM EDTA, 0.5 mM dithiotreitol, 1 mM phenylmethylsulfonyl fluoride, 10 μg/mL soybean trypsin inhibitor and 10 μg/mL leupeptin. The homogenate was centrifuged at 4 °C for 20 min at 24,000× *g*, and the supernatant was loaded into centrifugal concentrator tubes. The activity of XOR was determined in the ultrafiltered supernatant by a fluorometric kinetic assay based on the conversion of pterine to isoxanthopterine in the presence (total XOR) or absence (XO activity) of the electron acceptor methylene blue [61].

#### 4.2.4. Tissue Myeloperoxidase (MPO) Activity

The MPO activity was measured by the method of Kuebler et al. [62]. Briefly, the tissue was homogenized with Tris-HCl buffer (0.1 M, pH 7.4) containing 0.1 M polymethylsulfonyl fluoride to block tissue proteases, and then centrifuged at 4 °C for 20 min at 24,000× *g*. MPO activities of the samples were measured at 450 nm (UV-1601 spectrophotometer; Shimadzu, Kyoto, Japan), and the data were referred to the protein content.

#### 4.2.5. Tissue Nitrotyrosine (NT) Level

Free NT, as a marker of peroxynitrite generation, was measured by enzyme-linked immunosorbent assay (Cayman Chemical, Ann Arbor, MI, USA). Small intestinal tissue samples were homogenized and centrifuged at 15,000× *g*. The supernatants were collected and incubated overnight with antinitrotyrosine rabbit IgG and nitrotyrosine acetylcholinesterase tracer in pre-coated (mouse antirabbit IgG) microplates followed by development with Ellman’s reagent. NT content was normalized to the protein content of the bowel homogenate and expressed in ng/mg [63].

#### 4.2.6. Hydrogen Peroxide Production in Whole Blood

A 10 μL sample of whole blood and 50 μL zymosan were added to 1 mL Hank’s solution (PAA Cell Culture, Westborough, MA, USA) and the mixture was incubated at 37 °C for 30 min, until assay [64]. The chemiluminometric response was measured with a Lumat LB9507 luminometer (Berthold Technologies, Wildbad, Germany) during a 30-min period after the addition of 100 μL of lucigenin and luminol reagent.

#### 4.2.7. Laboratory Serum Diagnostics

Blood samples taken from the abdominal vena cava were analyzed for aspartate-aminotransferase (AST), alanine-aminotransferase (ALT) and lactate-dehydrogenase (LDH) using a fully automated clinical chemistry analyzer (Cobas 6000 automatic chemical analyzer, Roche Diagnostics, Budapest, Hungary).

#### 4.2.8. Quantitative and Qualitative Analysis of the Bowel Microbiome

Fecal samples were obtained from the terminal ileum of the animals (n = 6-6). Fresh stools were collected in a sterile stool container, which was immediately closed, and kept in a plastic zippered pocket until it was reopened. In this routine protocol, all manipulations (mixing, filtration, serial dilution and plating) were realized under sterile hood. For colony forming unit (CFU) counts, the samples were diluted serially in Lysogeny broth (LB) medium, serial dilutions ranging from 10^−1^ to 10^−10^ were performed on the stool samples, under both aerobic and anaerobic conditions. After homogenization, 10 μL of each dilution was plated on LB agar plates which were incubated for 48 h at room temperature. After incubation, the number of colonies were counted on the plate. We calculated the actual number of bacteria in the sample considering the dilution factor.

#### 4.2.9. Metagenomics

For total community DNA isolation 1 mL of pooled fecal samples (n = 6-6) was used. DNA extractions were carried out using the Zymo Research Fecal DNA kit (D6010, Zymo Research, Irvine, CA, USA). The quantity of DNA was estimated using a Qubit 2.0 Fluorometer (Life Technologies, Carlsbad, CA, USA). DNA purity was tested by agarose gel electrophoresis and on an Agilent 2200 TapeStation instrument (Agilent Technologies, Santa Clara, CA, USA).

Ion Torrent PGM Fragment libraries of 200 nt were generated from the pooled DNA (taken from the three parallel reactors) according to the appropriate protocols (Ion Torrent PGM, Life Technologies, Carlsbad, CA, USA). A total of 1 μg pooled total metagenomic DNA from each sample was used for library preparations. Ion Xpress Plus Fragment Library Kit was used. Adapter ligation and nick translation were performed by Ion Shear Plus Reagents Kit. Size selection was performed in 2% agarose gel, then library amplification was achieved by using Platinum PCR SuperMix. ION Library TaqMan qPCR was used for quantitation, and an Ion PGM 200 Xpress Template kit was used for the emulsion PCR. Sequencing was performed on an Ion Torrent Personal Genome Machine™ using Ion 318 chip. Between 200,000–300,000 high quality reads were generated for each sample.

MG-RAST (Metagenomics Rapid Annotation using Subsystem Technology) was used for phylogenetic and functional assessments. Taxonomical and functional assessment were performed after filtering the data. For the functional and taxonomical analyses MG-RAST utilized the M5nr protein database (GO, IMG, KEGG, NCBI (RefSeq and GenBank), SEED, UniProt, eggNOG and PATRIC) and various ribosomal RNA databases (RDP, Silva and Greengenes), respectively [65].

#### 4.2.10. In Vivo Microscopy

The extent of structural damage in the terminal ileum and distal colon was evaluated by CLSEM, developed for in vivo histology. The mucosal surface of the terminal ileum (5 cm proximal to the cecum) and mucosal surface of the distal (8 cm proximal to the anus) colon were surgically exposed and laid flat for examination. The analysis was performed twice, separately by two investigators (PH and GV). Records about the microvascular structure were taken after IV administration of 0.3 mL of fluorescein isothiocyanate-dextran (FITC-dextran 150 kDa, 20 mg/mL solution dissolved in saline, Sigma-Aldrich Inc., St. Louis, MO, USA). The objective lens of the device was placed onto the mucosal surface of the ileum and colon, confocal imaging was performed 5 min after dye administration (1 scan/image, 1024 × 1024 pixels and 475 × 475 μm per image). The changes in the mucosal architecture were examined following topical application of the fluorescent dye acriflavine (Sigma-Aldrich Inc., St. Louis, MO, USA). The surplus dye was washed off the mucosal surface of the colon with saline 2 min before imaging.

#### 4.2.11. Standard Histology

Samples from colon and terminal ileum were fixed in buffered formaldehyde solution (4%) and embedded in paraffin and 5-μm thick sections were stained with hematoxylin and eosin (H&E) and Periodic Acid-Schiff (PAS). Structural changes were assessed independently and blindly by two investigators (BeB and BáB). Histological scoring system (1–5 grade) was used to represent a composite number of villous atrophy (ileum) or crypt damage (colon), epithelial injury and inflammatory cellular infiltration (Table S1).

#### 4.2.12. Statistical Analysis

The statistical analysis was performed with SigmaStat 13.0 statistical software (Jandel Corporation, San Rafael, CA, USA). Changes in variables between groups were analyzed with one-way ANOVA followed by Holm–Sidak test. Correlation between variables was analyzed with Pearson’s product-moment correlation test. Data were expressed as means ± SEM. Histopathological changes were evaluated with ANOVA on Ranks followed by Tukey’s test. Data were expressed as median and minimum/maximum values. Values of *p* < 0.05 were considered statistically significant.

## Figures and Tables

**Table 1:** Serum necroenzyme levels. Aspartate-aminotransferase (AST), alanine-aminotransferase (ALT) and lactate-dehydrogenase (LDH) expressed in U/L. Data are presented as mean ± SEM. * *p* < 0.05 vs. control (one-way ANOVA, Holm–Sidak test) are considered as significant.

	AST (U/L)	ALT (U/L)	LDH (U/L)
Control	66.4 ± 8.5	101.8 ± 10.5	707.2 ± 271.9
Rifaximin	62.6 ± 8.6	110.2 ± 23.3	2025.6 ± 1331.5 *
Ceftriaxone	55.8 ± 13.6	96.0 ± 13.1	1258.8 ± 610.4 *
Combined	65.6 ± 5.3	101.0 ± 31.0	3571.2 ± 2094.3 *

## Data Availability

The data that support the findings of this study are available from the corresponding author upon reasonable request.

## Supplementary Materials

The following supporting information can be downloaded at: https://www.mdpi.com/article/10.3390/ijms23095064/s1.
