# Supplementary material for: Mitochondrial Side Effects of Surgical Prophylactic Antibiotics Ceftriaxone and Rifaximin Lead to Bowel Mucosal Damage
Source: Int J Mol Sci. 2022 May 3;23(9):5064. doi: 10.3390/ijms23095064 (PMC9103148; doi:10.3390/ijms23095064)
Supplement: Supplementary file 1 [file ijms-23-05064-s001.zip › ijms-1680306-supplementary.pdf]

Supplementary Materials

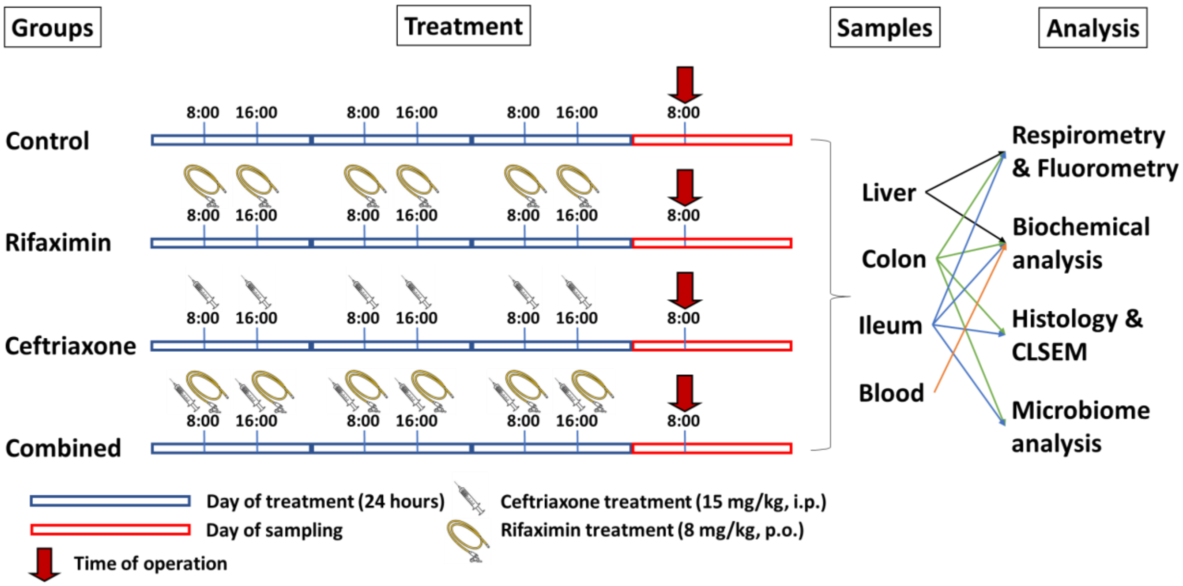

Figure S1. *In vivo* protocol for antibiotic treatment. Male Sprague-Dawley rats (300±25 g) were treated with Ceftriaxone injection (15 mg/kg *i.p.*), Rifaximin gavage (8 mg/kg *p.o.*), or the combination of both twice a day, for 3 consecutive days. Samples were taken 16 hours after the final treatment, on the following day. CLSEM: confocal laser scanning endomicroscopy, *i.p.*: intraperitoneal, *p.o.*: per os

Supplementary Materials

Ileum				
**Intestinal structure damage**	**Villous architecture**	**Inflammatory cell infiltrate**	**Inflammatory cell infiltrate extent**	**Surface epithelium**
**Grade 5 - Severe**	Total atrophy	Dense neutrophilic infiltrate	Transmural, serosal	Ulcerations
**Grade 4 - Marked**	Subtotal atrophy	Moderate neutrophil infiltrate	Transmural	Mucosal erosions
**Grade 3 - Moderate**	Partial atrophy	Scattered neutrophiles	Mucosal, submucosal	Goblet cell loss
**Grade 2 - Mild**	Villous blunting	Leukocytic infiltrate	Mucosal	Hyperplasia, apoptosis
**Grade 1 - Normal**	Normal	Normal	Normal	Normal

Colon				
**Intestinal structure damage**	**Villous architecture**	**Inflammatory cell infiltrate**	**Inflammatory cell infiltrate extent**	**Surface epithelium**
**Grade 5 – Severe**	Crypt abscess	Dense neutrophilic infiltrate	Transmural, serosal	Ulcerations
**Grade 4 - Marked**	Crypt loss	Moderate neutrophil infiltrate	Transmural	Mucosal erosions
**Grade 3 - Moderate**	Cryptitis	Scattered neutrophiles	Mucosal, submucosal	Goblet cell loss
**Grade 2 - Mild**	Crypt distortion	Leukocytic infiltrate	Mucosal	Hyperplasia, apoptosis
**Grade 1 - Normal**	Normal	Normal	Normal	Normal

Table S1. Histological scoring system (1-5 grade) to evaluate the morphological changes in the ileum and colon**.**
